# Physiological Responses of the Green Shore Crab, *Carcinus maenas*, During Acute and Chronic Low Temperature Exposure

**DOI:** 10.3390/ani14213049

**Published:** 2024-10-22

**Authors:** Molly L. Rivers, Cynthia H. McKenzie, Iain J. McGaw

**Affiliations:** 1 Department of Ocean Sciences, Memorial University of Newfoundland, 0 Marine Lab Rd., St. John’s, NL A1C 5S7, Canada; cynthia.mckenzie@dfo-mpo.gc.ca (C.H.M.); ijmcgaw@mun.ca (I.J.M.); 2 Northwest Atlantic Fisheries Centre, Fisheries and Oceans Canada, 80 East White Hills Rd., St. John’s, NL A1C 5X1, Canada

**Keywords:** dormancy, energy expenditure, heart rate, Newfoundland, oxygen consumption

## Abstract

The green shore crab (*Carcinus maenas*) is native to Western Europe but has spread around the globe and is described as one of the top 100 worst invasive species. On the east coast of North America, their northern-most limit is the island of Newfoundland, Canada, where they can experience water temperatures as low as −1 °C. We investigated the physiological responses of *C. maenas* to a temperature reduction regime as well as to long-term acclimation to temperatures representative of winter (2 °C) and summer (12 °C) in Newfoundland. Heart rate, oxygen consumption and estimated energy expenditure declined steadily with decreasing temperature, but a marked change was observed between 6 and 4 °C, with lowest levels recorded in 2 °C. After long-term acclimation to 2 °C there was a sustained reduction in physiological parameters. Even though these physiological parameters were very low in 2 °C, the crabs still exhibited intermittent activity. This supports the presence of a dormancy, rather than true torpor/hibernation below 5 °C, in which crabs will continue to actively move and feed, albeit much more slowly. The population in Newfoundland contains haplotypes from both the invasive northern and southern lineages, and they appear to retain a similar low temperature response compared with most other populations of green crab from both their native and expanded range.

## Simple Summary

*Carcinus maenas* is native to NW Europe but is an important invasive species worldwide. On the island of Newfoundland, Canada, this species experiences the coldest winter-time temperatures compared to either their native or any other area of their invaded range. During a temperature reduction (12 to 2 °C) *C. maenas* exhibits a decline in heart rate, oxygen consumption and energy expenditure with a noticeable drop between 6 and 4 °C. After long-term acclimation to 2 °C physiological parameters remain routinely low. However, *C. maenas* still exhibits some activity, suggesting they enter a dormancy at temperatures between 6 and 4 °C rather than entering a true torpor or hibernation.

## 1. Introduction

The green shore crab (*Carcinus maenas*) is a small decapod crustacean belonging to the family *Portunidae.* This species reaches a maximum carapace width of approximately 10 cm [1] and has an average lifespan that varies from 3–7 years, depending on geographical location [2]. They inhabit sheltered bays with soft sediment, the rocky shore intertidal zone and estuarine habitats [2,3,4]. *C. maenas* is native to Northwest Europe and Northern Africa, but over the last 200 years have expanded their range to include every continent, except Antarctica [3,5]. The main mechanism for their global dispersal is thought to be maritime activity, specifically in ballast water of vessels as larvae/juveniles [5,6]. To date, they have established populations on the East coast of North America in 1817 [7,8], Australia in the 1800s [9], South Africa in 1983 [10,11], Japan in 1984 [12], the West coast of North America in 1989 [8,13] and in Argentina in 2003 [14].

*C. maenas’* success as an invader is in part due to their broad thermal tolerance [15]. Adult *C. maenas* can survive in temperatures up to approximately 38 °C [16], but tend to avoid water temperatures above 28 °C [17]. Information on their lower thermal tolerance is limited, but they can tolerate acute exposure to temperatures as low as −1 °C [16,18,19]. Their larvae, however, have a narrower temperature tolerance and require water temperatures to remain above 10 °C for several months for successful development to occur [14,20,21]. Although *C. maenas* continue to invade new locations and expand their range within established locations, they have not colonised polar or tropical regions, most likely due to the temperature limitations of their larvae. Minimum seasonal sea surface temperature has been suggested as a highly influential factor in determining northern range expansion and limitation of green crabs [14,22,23]. Thermogeographic models predicting their future range expansion suggest invasion potential depends on water temperature and origin of the population (i.e., Southern or Northern Europe) [24].

The physiological responses of *C. maenas* to temperature increase has been extensively studied [25,26,27,28]. Heart rate and oxygen consumption are frequently used as indicators for thermal tolerance in ectotherms [29]. Heart rate increases with temperature until a critical thermal maximum temperature (CT_max_) is reached, which is estimated to be between 30 °C and 38 °C for *C. maenas* [25,30,31,32,33]. The exact temperature at which CT_max_ occurs is dependent on the rate of temperature change, prior acclimation temperature, and geographical origin [16,32,34,35,36]. Heart rate becomes erratic and declines rapidly after CT_max_ is reached [30,37]. Oxygen consumption (MO_2_) follows a similar pattern increasing steadily until CT_max_ is reached, with a sharp decline thereafter [27,38,39,40].

In comparison to temperature increases, there has been much less work on the physiological responses of *C. maenas* to declining temperatures [16,19]. A significant reduction in both heart rate and metabolism occurs at 5 °C suggesting a state of torpor may occur at these temperatures [16,41,42,43]. Only a few articles have investigated responses below 5 °C, and while these do show *C. maenas* can survive acute exposure to temperatures as low as −1 °C, [16,28], there is no information on the effects of prolonged cold exposure on the physiological responses of this species.

*C. maenas* were first reported in Placentia Bay, on the island of Newfoundland (NL), Canada, in 2007 [44,45]. The population in Placentia Bay are a hybrid group containing a mix of haplotypes from the southern lineages (originally settling in New England) and a more recent cold-tolerant northern European lineage [23,46,47]. This hybrid population have since expanded their range westward along the southern coast, with a separate introduction (northern lineage) on the west coast of NL which has expanded along the western coast to the Northern Peninsula of NL [4,45]. Water temperatures in coastal regions of southern NL average approximately 0 to 2 °C during the winter; these are harsher conditions than experienced by *C. maenas* in the entirety of their current (native and invasive) range [24,48,49]. Indeed, the invasion of NL by *C. maenas* defied thermogeographic models as the minimum sea surface temperatures during the winter was predicted to be too low for larval survival [24]. Despite this, very little is known as to how this hybrid population survives during the low winter temperatures characteristic of NL waters. In their native range *C. maenas* move from the intertidal and shallow subtidal zone into deeper warmer waters during the winter [50,51]. However, our recent work with the NL population shows that although they do retreat from the intertidal zone, they tend to remain within the shallow coastal bays during the winter where they are exposed to temperatures between 0 and 2 °C for several months [52]. This is interesting because very little is known about the long-term seasonal responses to cold temperatures of *C. maenas* in their native range, let alone the hybridized NL population which appear to be on their northern limits of cold tolerance. Thus, the aims of the present experiment were to investigate the physiological responses of *C. maenas* to the winter-time sea temperatures in NL (here tested at 2 °C). Heart rate and oxygen consumption were measured in response to an acute decline in temperature and after long-term exposure (>2 months) to cold water temperatures.

## 2. Materials and Methods

### 2.1. Specimen Collection and Housing

Intermoult adult green crabs (*Carcinus maenas*) were collected between June and October 2019 and 2020 using baited net traps set at multiple locations in northern Placentia Bay, NL. Large males (carapace width > 5 cm) were brought back to the Ocean Sciences Centre, Memorial University. Female crabs were not used in experiments because protocols to prevent spread of this invasive species precluded their transport and live storage. The crabs were held in flow-through seawater (31–32 ppt) tanks at ambient temperatures ranging between −0.5 and 13 °C. Air stones in each tank maintained the oxygen concentration above 90% saturation. Cylindrical PVC pipes (10 cm diameter × 12 cm or 24 cm length) were added to the tanks to provide shelter. The crabs were fed herring once a week, and any dead specimens and uneaten fish were promptly removed from tanks to minimize fouling of the water.

Before being used in experiments, individual crabs (40–87 g) were labelled with a foam tag glued to the dorsal surface of the carapace and their wet weight (g) and carapace width (mm) were recorded. The crabs were then moved into the laboratory and acclimated in 45 L flow-through seawater tables maintained at 12 °C for a minimum of 2 weeks prior to the first set of experiments [35]. This temperature represents the average summer sea surface temperatures in Placentia Bay, NL [49]. To minimize disturbance and eliminate any diurnal rhythms, constant red light was maintained in the lab as crustaceans are minimally affected by these wavelengths [53] (Cronin, 1986). Black plastic screens were hung around the tanks to prevent visual disturbance to the animals. Crabs were starved for 3–5 days prior to trials to ensure they were in a post-absorptive state [54] and were placed in the experimental apparatus and allowed to settle after handling for >12 h before recording began [55].

### 2.2. Temperature Reduction Experiments

In the first experimental series, physiological responses (heart rate, treatment: n = 9, control: n = 15, oxygen consumption (MO_2_) treatment: n = 16, control: n = 10) were assessed during an incremental temperature reduction regime. Crabs that had previously been acclimated to 12 °C were placed into the experimental apparatus at a starting temperature of 12 °C and recording began following an initial 12 h settling period in the apparatus. Data were collected at hourly intervals for 24 h, at 12 °C, the temperature was then lowered by 2 °C (over approximately 30 min), and data were recorded for a further 24 h at 10 °C (no data collection occurred during the 30 min periods of temperature change). This process was repeated until the temperature reached 2 °C. The minimum test temperature used in the present study was 2 °C as the winter water temperature in Newfoundland remains at approximately 2 °C, and only drops below 0 °C for ≤1 month during the winter [53]. For each experiment, control trials were carried out at 12 °C for 6 consecutive days (time-period for the temperature reduction) to rule out time as a causal factor. Individual crabs were only used once during temperature treatment or control experiments.

### 2.3. Long-Term Acclimation to Winter and Summer Time Temperature Experiments

In a second series of experiments, physiological responses were monitored at a constant temperature (either 12 °C or 2 °C) for 6 days following acclimation of approximately 2 months to each respective temperature. This experiment mimicked the prolonged temperature conditions that *C. maenas* experience during the summer and winter months in NL. The following number of animals were used in each experimental treatment: heart rate (12 °C: n = 15, 2 °C: n = 10); MO_2_ (12 °C: n = 16, 2 °C: n = 18); data from each 6 day experimental period were analysed in 24 h blocks.

### 2.4. Heart Rate

To measure heart rate (HR), Newshift infrared heart rate monitors (Leiria, Portugal) were attached to the carapace of each crab directly above the heart using dental wax and super glue. Crabs were then placed in individual perforated plastic boxes (18 cm × 18 cm × 7 cm depth) and held in a flow through seawater table. They were allowed to settle for at least 12 h before recording began. Each heart rate monitor was attached to a Newshift AMP03-U heart rate amplifier (Leiria, Portugal) with the continuous output recorded using ADInstruments LabChart7 software (Colorado Springs, CO, USA). The holding containers were large enough for even the largest crabs to fit comfortably, allowing the animal to turn while preventing excessive movement which can cause increases in HR [35,56,57]. The mean HR for each 24 h period was calculated by measuring the HR during the first minute (or the closest minute in which no pause in heart rate was occurring) of each hour throughout the trial. HR was not calculated during the first hour after the temperature changeover because crabs usually react with a startle response which would artificially inflate rates [35,57].

### 2.5. Metabolic Rate and Energy Expenditure

An L-DAQ intermittent flow respirometry system (Loligo systems, Viborg, Denmark) was used to measure oxygen consumption (mg O_2_ kg h^−1^). Individual crabs were placed in separate cylindrical chambers (10 cm diameter × 8 cm height). Each chamber was equipped with two seawater pumps; the first pump continually flushed water through the chamber to maintain oxygen saturation during non-measurement periods. During the measurement periods, this pump is turned off and the chamber is sealed while the second pump recirculates the water within the chamber (10 L min^−1^) [27]. The respirometry chambers were fitted with fibre optic oxygen probes that measured oxygen saturation within the chamber during the measurement periods, calculating the metabolic rate (MO_2_). This fully automated system was set to recirculate water within the chambers for 40 min (measurement period) and to flush for 20 min every hour. This cycle was repeated for the duration of each trial (6 days). During the long-term acclimation trials at 2 °C MO_2_ was very low, so the recirculation period was increased to 50 min (and the flush cycle decreased to 10 min) to allow for a measurable decline in MO_2_ within the chamber. Data were recorded on a Loligo data acquisition system (Copenhagen, Denmark) and using Loligo Systems AutoResp4 software (Viborg, Denmark), MO_2_ (mg O_2_ kg h^−1^) was calculated at hourly intervals resulting in 24 MO_2_ measures per specimen per day. The mean, maximum (the highest value recorded), and resting (calculated as the average of the lowest five MO_2_ values) MO_2_ values for every 24 h period were also calculated. The estimated energy expenditure of each animal was calculated from the total MO_2_ over each 24 h period as a function of the mass of each crab (using KaleidaGraph V5 software). This value was standardized to kJ using the conversion factor of 1 mg O_2_ consumption = 0.014 kJ [58].

### 2.6. Statistical Analysis

To test for differences in heart rate, MO_2_ and energy expenditure, for both the temperature reduction and long-term temperature acclimation trials, two-way ANOVAs were performed (in IBM SPSS) adding time as a repeated measures factor (as the same individual was measured across the duration of the experiment). Post-hoc pairwise comparisons were performed using Fisher’s Least Significant Difference (LSD) test. For the long-term acclimation trial, time was included as a within-factors subject to account for the repeated measures and to rule out time as a causal factor. In each case the degrees of freedom for each test are given in subscript after the F value.

## 3. Results

### 3.1. Temperature Reduction

**Figure 1:**
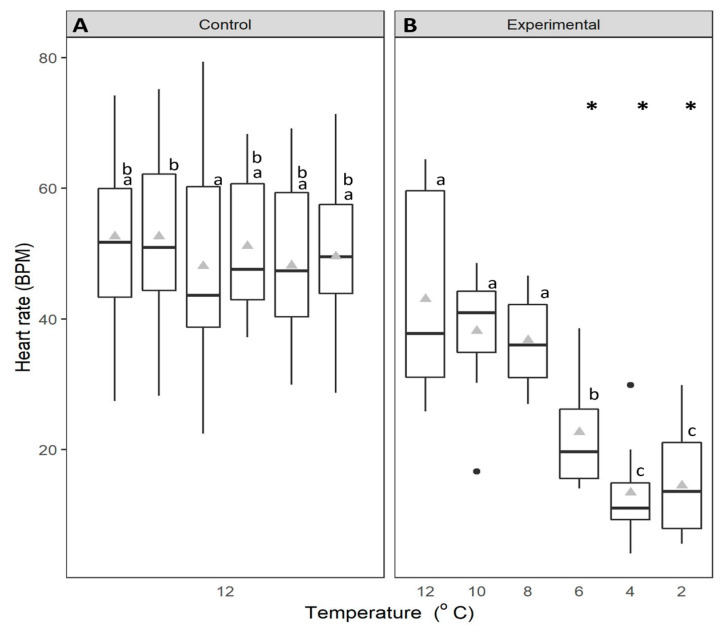
Heart rate (beats per minute) of green crabs (control n = 15, experimental n = 9). (**A**): control crabs, held at 12 °C for 6 days and (**B**): experimental crabs, experienced a reduction in temperature from 12 °C to 2 °C. The data are displayed for each 24-h period over 6 days. Whiskers represent 95% confidence limits, boxes show upper and lower quartiles, horizontal bar presents median values, black circles denote outlying values and grey triangles denote the mean. * denotes significant changes in the temperature treatment relative to the control. Lowercase letters (above each bar) denote significant differences within treatment groups, like letters are not significantly different from one another.

Heart rate was significantly affected by the interaction between time and treatment type (mixed factorial ANOVA: F_5,110_ = 15.889, *p* < 0.001). Heart rates for the control group (held at 12 °C) remained steady (Fisher’s LSD *p* > 0.05) between mean levels of 48 ± 3.35 BPM and 53 ± 2.93 BPM during the 6-d experimental period (Figure 1A). In contrast, during the temperature reduction treatment, heart rate decreased significantly from mean rates of 43 ± 5.19 BPM at 12 °C to 14 ± 2.79 BPM at 2 °C (Fisher’s LSD *p* < 0.05) (Figure 1B). There was no difference in heart rate between temperatures of 12, 10 and 8 °C, however, a marked reduction occurred at 6 °C where heart rate declined, on average, by 14 BPM (Fisher’s LSD, *p* < 0.05). The heart rates measured at 6 °C and below were significantly lower than those of the control group and at 8 °C and above. At temperatures of 4 and 2 °C the heart rate was similar between test organisms. Neither maximum nor minimum heart rate at 12 °C was significantly different between the control group and the treatment group at 12 °C (max: 79 BPM and 64 BPM, respectively; min: 26 BPM and 27 BPM, respectively) (Figure 1).

**Figure 2:**
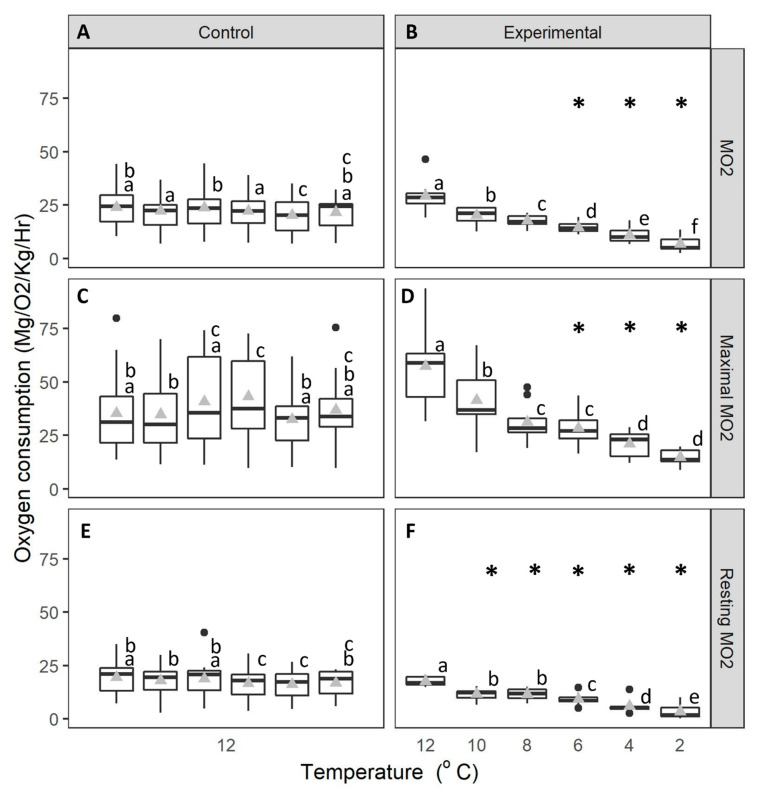
Oxygen consumption (mg/O_2_/kg/h) of green crab (control n = 16, experimental n = 10). (**A**): MO_2_ of control crabs (held at 12 °C), (**B**): MO_2_ of experimental crabs (experiencing a reduction in temperature from 12 °C to 2 °C), (**C**): maximal MO_2_ of control crabs, (**D**): maximal MO_2_ of experimental crabs, (**E**): resting MO_2_ of control crabs and (**F**): resting MO_2_ of experimental crabs and control crabs, held at 12 °C. The data are displayed for each 24-h period over 6 days. Whiskers represent 95% confidence limits, boxes show upper and lower quartiles, horizontal bar presents median values, black circles denote outlying values and grey triangles denote mean. * denotes significant changes in the temperature treatment relative to the control. Lowercase letters (above each bar) denote significant differences within treatment groups, like letters are not significantly different from one another.

During the control experiment, the mean MO_2_ (Figure 2A), as well as maximal (Figure 2C) and resting MO_2_ (Figure 2E), remained stable over the 6-d trial period (mean value: 20 ± 2.08–24 ± 2.50 mg O_2_/kg/h for MO_2_, 32 ± 3.58–43 ± 5.23 O_2_/kg/h for maximal MO_2_, and 16 ± 1.62–20 ± 1.98 mg O_2_/kg/h for resting MO_2_ (Figure 2)). In contrast, these values all decreased when temperature was lowered from 12 °C to 2 °C (mean values: 29 ± 2.26–7 ± 1.75 mg O_2_/kg/h for MO_2_, 57 ± 6.06–15 ± 1.22 O_2_/kg/h for maximal MO_2_, and 18 ± 0.71–3 ± 1.07 mg O_2_/kg/h for resting MO_2_) (Figure 2B,D,F). In addition, the mean, maximal and resting MO_2_ were all significantly affected by the interaction between temperature and time (mixed effects ANOVAs, MO_2_: F_5,120_ = 35.097, *p* < 0.001; maximal MO_2_: F_5,120_ = 15.971, *p* < 0.001; resting MO_2_: F_5,120_ = 16.572, *p* < 0.001), showing a decline in line with decreasing temperature. MO_2_ and maximal MO_2_ became significantly different to the control group at and below 6 °C, while resting MO_2_ was significantly different at and below 10 °C (Fisher’s LSD, *p* < 0.05 Figure 2).

**Figure 3:**
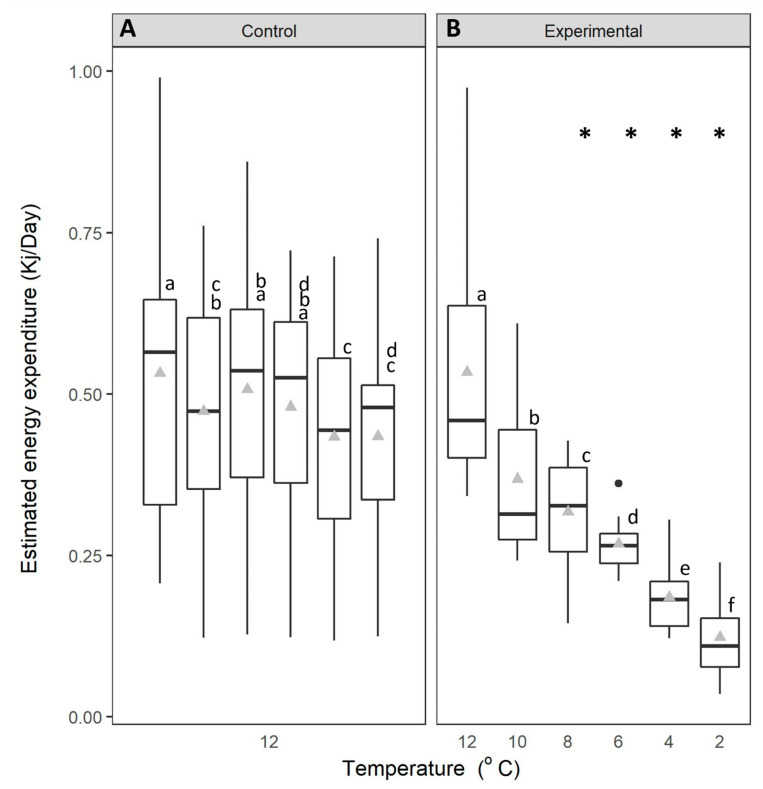
Estimated energy expenditure (kJ/day) of green crabs. (**A**): control crabs, held at 12 °C and (**B**): experimental crabs, experiencing a reduction in temperature from 12 °C to 2 °C. The data are displayed for each 24-h period over 6 days. Whiskers represent 95% confidence limits, boxes show upper and lower quartiles, horizontal bar presents median values, black circles denote outlying values and grey triangles denote mean. * denotes where we observed significant differences in temperature treatment relative to the control. Lowercase letters (above each bar) denote significant differences within treatment groups, like letters are not significantly different from one another.

The estimated energy expenditure (EEE) of crabs was significantly affected by the interaction between time and temperature (mixed effects ANOVA: F_5,120_ = 13.216, *p* < 0.001; Figure 3). The EEE of crabs in the control treatment (12 °C for 6 days) was maintained between mean levels of 0.43 and 0.53 ± 0.046 kJ/day (Figure 3A) during the 6-d experimental period (Fisher’s LSD, *p* > 0.05). In contrast, the estimated energy expenditure of crabs exposed to a reduction in temperature gradually declined with decreasing temperature (Figure 3B; Fisher’s LSD, *p* < 0.05). The EEE was significantly reduced in comparison to control individuals (Figure 3A) held at 12 °C at temperatures of 8 °C and below (Fisher’s LSD, *p* < 0.05).

### 3.2. Long-Term Acclimation to Winter and Summer Time Temperatures

**Figure 4:**
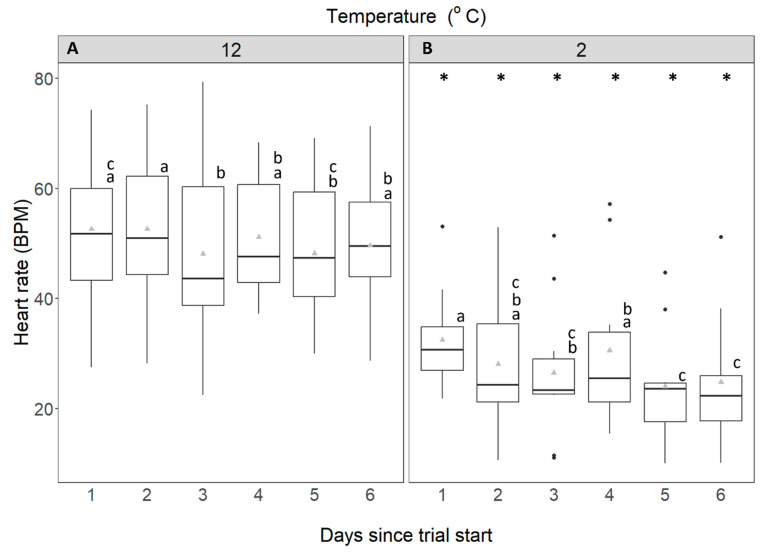
Heart rate (beats per minute) of *C. maenas*. (**A**): control crabs, held at 12 °C, and (**B**): experimental crabs, held at 2 °C. These data are displayed for each 24-h period over 6 days. Whiskers represent 95% confidence limits, boxes show upper and lower quartiles, horizontal bar presents median values, black circles denote outlying values and grey triangles denote mean. * denotes where heart rate is significantly different from that at control temperature. Lowercase letters (above each bar) denote significant differences within treatment groups, like letters are not significantly different from one another.

The heart rate of *C. maenas* acclimated to 12 °C ranged between mean values of 48 and 53 ± 13 BPM over the 6-d measurement period (Figure 4A). These values were significantly higher than 24 to 32 ± 12 BPM measured for crabs acclimated to 2 °C (mixed factorial ANOVA: F_1,23_ = 23.807, *p* < 0.001; Figure 4B). Heart rate was affected by the interaction between temperature and time, although temperature remained constant throughout. This difference is explained by the 2 °C acclimated group, which showed a significant reduction in mean heart rates on days 5 and 6 (Figure 4B; Fisher’s LSD, *p* < 0.05).

**Figure 5:**
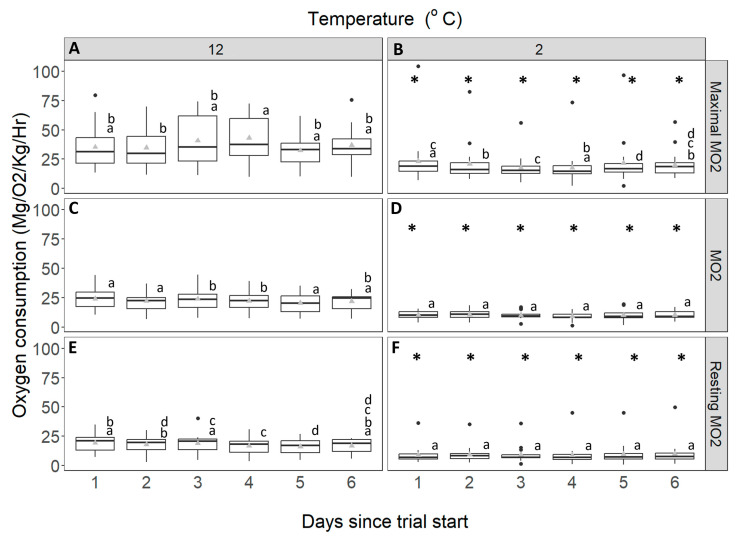
Oxygen consumption (mg/O_2_/kg/h) of *C. maenas*. (**A**): max MO_2_ of control crabs (held at 12 °C), (**B**): max MO_2_ of experimental crabs (held at 2 °C), (**C**): mean MO_2_ of control crabs, (**D**): mean MO_2_ of experimental crabs, (**E**): resting MO_2_ of control crabs and (**F**): resting MO_2_ of experimental crabs, control crabs, held at 12 °C. The data are displayed for each 24-h period over 6 days. Whiskers represent 95% confidence limits, boxes show upper and lower quartiles, horizontal bar presents median values, black circles denote outlying values and grey triangles denote mean. * denotes significant differences in MO_2_ from that at control temperature. Lowercase letters (above each bar) denote significant differences within treatment groups, like letters are not significantly different from one another.

Overall, the mean, maximal and resting MO_2_ were significantly lower at 2 °C compared with 12 °C (Figure 5) (mixed factorial ANOVA: max: F_1,33_ = 11.113, *p* = 0.002, mean: F_1,33_ = 10.376, *p* = 0.003, resting: F_1,33_ = 44.531, *p* < 0.001). The MO_2_ for any 24-h period at 12 °C varied between 20 and 24 mg O_2_/kg/h, while at 2 °C it varied between 11 and 13 mg O_2_/kg/h (Figure 5A,B). There was a significant interaction between time and temperature on MO_2_ (mixed factorial ANOVA: F_5,160_ = 2.908, *p* = 0.039). This interaction occurred because the 12 °C acclimated group showed a slight decrease in MO_2_ (Fisher’s LSD, *p* < 0.05), declining from 24 mg O_2_/kg/h on day 1 to 21.6 mg O_2_/kg/h on day 6, while the MO_2_ of 2 °C acclimated crabs remained stable throughout the 6-d experimental period (Figure 5B). Maximal MO_2_ was affected by the interaction between time and temperature (mixed factorial ANOVA: F_5,160_ = 3.729, *p* = 0.017) due to the maximal MO_2_ being more variable over time at 12 °C than at 2 °C (Figure 5C,D). The resting MO_2_ was not significantly affected by time or its interaction with temperature (mixed factorial ANOVA; interaction: F_5,160_ = 2.587, *p* = 0.066 time: F_5,160_ = 1.968, *p* = 0.132) (Figure 5E,F).

**Figure 6:**
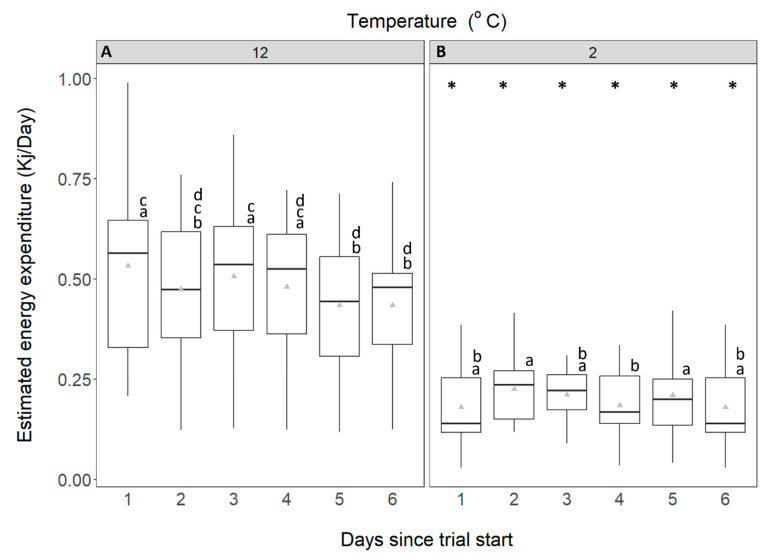
Estimated energy expenditure (kJ/day) of *C. maenas*. (**A**): control crabs, held at 12 °C and (**B**): experimental crabs, held at 2 °C. The data are displayed for each 24-h period over 6 days. Whiskers represent 95% confidence limits, boxes show upper and lower quartiles, horizontal bar presents median values, grey triangles denote mean. * denotes where energy expenditure is significantly different from that at control temperature. Lowercase letters (above each bar) denote significant differences within treatment groups, like letters are not significantly different from one another.

Estimated energy expenditure of *C. maenas* acclimated to 12 °C (0.48 kJ/h) was more than double that of the crabs acclimated to 2 °C (0.20 kJ/h) (mixed factorial ANOVA: F_1,32_ = 39.407, *p* < 0.001; Figure 6A,B). Time also affected energy expenditure (mixed factorial ANOVA: F_5,160_ = 4.216, *p* = 0.008); crabs in the 12 °C acclimated group exhibited lower energy expenditure on days 5 and 6 (Fisher’s LSD, *p* < 0.05). EEE of crabs acclimated to 2 °C remained stable between 0.18–0.22 kJ/h. In addition, mean energy expenditure at 12 °C was more variable than that measured at 2 °C (Figure 6A).

## 4. Discussion

### 4.1. Reduction in Temperature

The over-wintering temperatures in NL are some of the coldest experienced by *Carcinus maenas* throughout their global range [24,48,49]. *C. maenas* in southeastern NL (Placentia Bay and Fortune Bay) are a hybridized population containing haplotypes from both the northern and southern lineages [23,46,47,59]. It has been suggested that the ‘northern lineage’, originating from north-west Europe and Scandinavia, have greater cold tolerance than the ‘southern lineage’, originating from South Western Europe and Northern Africa [24,60,61]. However, these lineage-specific tolerances are based on observed genomic spatial structure and have not been experimentally validated [23,62,63].

When exposed to a decline in water temperature, *C. maenas* exhibited a reduction in metabolic parameters (heart rate, oxygen consumption, estimated energy expenditure). Both the heart rate and oxygen consumption of crustaceans are positively correlated with locomotor activity [63,64,65]. Overall, these results suggest that temperatures between 4 and 6 °C represent an important switch-over range for *C. maenas*. This inference is supported by the findings of [20] (which investigated the southern lineage population) that *C. maenas* enter a torpor-like state at approximately 5 °C. Torpor in endotherms is an energy conservation response to cold temperatures, in which reduced heart rate, metabolic rate and locomotor activity occurs [66]. Ectotherms body temperature changes directly in-line with water temperature, thus they experience a natural decline in physiological and behavioural reactions with water temperature [67]. *C. maenas*, however, also arouse rapidly and feed, albeit at a lower rate, and can make extensive movements, even during the winter [52]. Therefore, one should be careful classifying this decline in physiology as a true torpid state or hibernation which primarily applies to endotherms that actively downregulate body temperature, and in a few ectotherms such as the cunner fish (*Tautogolabrus adspersus*). Cunner completely cease locomotor activity and feeding during the winter, and downregulate their resting metabolic rate and cardiac output [68,69,70]. There was no evidence of this in *C. maenas* and parameters appeared to be reduced simply because of a lower metabolism at colder temperatures [52]. It might be more accurate, therefore, to describe it as a dormancy or dormant-like state to differentiate it from true torpor.

The rate of temperature decline in the temperature reduction experiments was 2 °C per day. This is faster than would be experienced during the seasonal change from summer to winter in Placentia Bay, NL, which varies on average by 10 °C. Most articles investigating responses of crustaceans to temperature use much more rapid temperature changes (>1 °C/h) and tend to investigate temperature increases [26,71,72,73]. Of the few studies investigating the response to declining temperature, this regime is the most representative of seasonal temperature change, as most other researchers have used much more drastic and rapid reductions in temperature [28,43]. Thermal equilibrium rates in aquatic crustaceans (time taken for core body temperature to match that of external temperature upon change) are approximately 5 min, which would mean that even in the rapid temperature change experiments (>1 °C/h) the body temperature would have equilibrated with the environment [74]. However, heart rate and neural signal response times decrease with increased rate of change, until a threshold rate of 2.5 °C per minute is reached [75]. Additionally, behavioural responses are also affected by the range of temperature change, with greater ranges of change causing larger but slower responses [19]. Similarly, movement activity of crayfish (*Astacus astacus*) has been shown to be most stable during an intermediate temperate range (10–19 °C) and is more impacted by similar degrees of change at either end of its temperature range (6–10 °C and 19–24 °C) [76,77]. Although body temperature equalizes rapidly, biochemical and neural, and thus physiological, reactions may be slower to adjust to temperature change, therefore, the slower rate of temperature change used in these experiments would likely produce more accurate results.

Although the experimental regime used here encompassed the full temperature change experienced by *C. maenas* in situ in coastal waters in Newfoundland, and the change was slower than in previous studies, it was still much more rapid than the typical seasonal changes. Therefore, we also investigated the responses of *C. maenas* after long-term acclimation to typical winter (2 °C) and summer (12 °C) temperatures in NL.

### 4.2. Long-Term Acclimation to Winter and Summer Temperatures

After long-term acclimation (>2 months) to cold temperatures (2 °C), *C. maenas* showed a reduction in heart rate, oxygen consumption and estimated energy expenditure. However, locomotor activity and feeding does not completely cease, indicating they maintain some responsiveness to their environment [52]. This is in line with previous studies showing green crabs have highly reduced movement below 5 °C, with complete inactivity around 0 °C [19,78]. The minimum test temperature used in the present study was 2 °C as the winter water temperature in Newfoundland remains at approximately 2 °C, and only drops to −0.5 °C for ≤1 month during the winter [52]. In addition, there were logistical problems to keeping water at 0 °C for extended periods of time in the lab. Although *C. maenas* in NL appear to undergo a dormancy with a significant reduction in metabolic parameters between 6 and 4 °C, they do not undergo a complete shut-down as occurs in some animals in the region, such as the cunner (*Tautogolabrus adspersus*), even after exposure to months of cold water. They do remain responsive to their environment, which could allow for opportunistic feeding or predator evasion [52].

Regarding acclimation time, the time animals are held at test temperature for the days to weeks before experiments commence is an important factor that influences physiological responses [79,80]. An acclimation time of approximately four weeks has been recommended for experiments using *C. maenas* [30,81]. After long-term exposure of >2 months to 2 °C, heart rate declined even during the 6-d test period. In contrast, the control animals held at 12 °C for the same 6-d period did not show any change over time. This could indicate that longer acclimation times are required at cold temperatures, and this could be more impactful on HR which is variable at best [35], as MO_2_ and EEE did not show this same trend.

Regarding settling time, the time animals spend in their test apparatus before experiments commence is also highly influential. Matveev and McGaw [80] recommend a minimum of 16 h settling time for use of *Cancer irroratus* in feeding behaviour trials. Experiments on handling stress of *C. maenas* suggest a settling time of ≥12 h is needed to eliminate the impact of handling [55]. These experiments were conducted at 10–12 °C, and so represent the ability of decapods to deal with environmental change when at normal thermal conditions. Experiments investigating physiological responses of decapods report settling times of approximately 12 h [17,82,83]. Our results, however, show heart rate declines from a mean of 32 BPM to 25 BPM over the course of the experiment at 2 °C, which could question whether longer settling times are required when using low acclimation temperatures. These results also support the idea that the crabs are dormant, or in a torpor-like state, rather than undergoing complete torpor/hibernation, per some anecdotal evidence from local fish harvesters.

An unexpected, but consistent, trend across all the traits was a greater inter-individual variation in warmer temperatures. For example, EEE at 12 °C had a range more than double that at 2 °C. Inter-individual variation in metabolic rate might reflect variations in “personality” (defined as consistent individual differences in behavior, with bold and shy individuals), which are known to occur across multiple species [84,85,86,87,88,89]. Such variation in personality within populations has been observed to impact the response of labile traits to temperature changes [87]. The presence of personalities and the impact of inter-individual variation on the response to environmental change in crustaceans has not been studied in detail. The results presented here suggest that the physiological requirements to reduce energy usage and undergo dormancy after prolonged exposure to highly reduced temperatures cause inter-individual variation observed in warmer temperatures to be reduced. However, this study does not investigate consistent inter- or intra-individual differences with temperature, and, as such, this high degree of variation seen at 12 °C could be incidental, thus caution should be used when interpreting these results.

## 5. Conclusions

These experiments indicated that *C. maenas* showed a significant reduction in physiological responses at water temperatures between 6 and 4 °C. These responses to changing temperature seen here by *C. maenas* from the southeastern coast of NL are similar to those exhibited by other populations of *C. maenas* in both their native and invasive ranges [16,19,20,41,42,43,90]. This suggests that the hybridized NL population retains a similar temperature induced dormancy response to low temperatures with most other populations of green crabs, therefore, at least for the adult stages, this may be inherent across populations.

## Data Availability

Data can be made available upon request and at the discretion of the authors.
